# Genito-Urinary and Syphilitic Diseases

**Published:** 1897-03

**Authors:** Byron H. Daggett

**Affiliations:** Buffalo, N. Y.


### GENITO-URINARY AND SYPHILITIC DISEASES.

CONDUCTED BY BYRON H. DAGGETT, M. D., Buffalo, N. Y.

### LOCAL ANESTHESIA BY EUCAINE.

LEQUEU (Association Francase d' Urologie ; *The Universal Medical Journal*, November, 1896,) claims, as a result of his experience, that eucaine is equal, if not superior, to cocaine for producing anesthesia of the urinary apparatus, that it is less toxic and more stable than cocaine, and that its solution may be sterilised by boiling. It is less poisonous than cocaine, since 1¼ grains of eucaine caused the death of a guinea-pig in one and a half hours, while 9-10 of a grain of cocaine killed a guinea-pig, of the same weight, in three-quarters of an hour. He uses a 1 per cent. solution, and injects into the urethra or under the skin ¾ or 9-10 of a grain, and follows the rules given by Réches for using cocaine, and alleges that he has never observed any of the accidents attending the primary use of cocaine.

In cystoscopy he injects 160 to 200 grammes (5⅓ to 6⅔ ounces) of a 5 per cent. solution into the bladder. Eucaine congests, cocaine decongests. For this reason cocaine should be used in cases in which there is pronounced bleeding.

Carlier called attention to the fact that sterilised cocaine solutions may be prepared as follows : cocaine is put up in packages of sterilised paper, and at the time of the operation the contents are poured into sterilised water. In this connection Carlier also mentions a special method for producing complete anesthesia of the penis. This method consists in making four injections around the base of the penis.

---

### URETHRAL ELECTROLYSIS.

BISHOP (American Electrotherapeutic Association, September 30, 1896,) claims that the Newman treatment of urethral stricture by electrolysis possesses many advantages over other methods. Sir Henry Thompson said that an organic stricture is a permanent condition, and cannot be entirely dissipated by any known means. Dilate it, cut it, divulse it, "but then, more or less, the morbid elements must always remain. When a man once has organic stricture, he has it forever."

The advantages of electrolysis are : (1) it is practically painless ; (2) it is not in the least bit dangerous ; (3) it is seldom contraindicated ; (4) it never makes false passages ; (5) it attends to the stricture only ; (6) it appeals to the reason of the patient and gains his confidence ; (7) it cures.

Dr. Bishop alleges that he has never seen or heard of any ill-effects following the skilful and careful use of electrolysis, while nervous chills, urethral fever, are not uncommon accompaniments of other surgical procedures in the urethral canal. The doctor further alleges that there is abundant evidence, given by eminent men, to prove conclusively that the Newman method of electrolysis cures absolutely.

Causes of failure to get good results : (1) clumsiness in handling instruments ; (2) too much current or too long application ; (3) too much pressure on electrode ; (4) too frequent operations ; (5) too much anxiety ; (6) faulty instruments ; (7) faulty methods of applying the current ; (8) the use of the wrong current.

---

Arnozan (Medical Congress at Nancy, August, 1896,) said the urinary albuminoids are peptones, propeptones, serum albumin, globulin, nucleo-albumin and perhaps others. Albumin has been found in the urine of many persons presenting the appearance of health, but never in normal urine. Its presence signifies impairment and vulnerability, and its prognosis is not yet established. Slowing of the renal circulation, alterations in the blood and lesions of the filtering membrane produce albuminuria. Blood changes may be first in date, and the albuminuria is alleged to be depurative. Albumin is found in the exudates of all inflamed mucous membranes, and is an inflammatory exude in epithelial lesions of the kidney. The casts and chemical composition of the urine are of more value in making a prognosis than the abundance and variety of the albumins ; the state of the renal function is of far greater importance than the amount of albumin.

Albuminuria always indicates a vascular or nutritive disturbance of the kidney, and frequently ends in Bright's disease. Albuminuria complicates many severe maladies and renders prognosis more unfavorable. If it continues after the subsidence of severe fevers and the disappearance of pathogenic germs in the urine, the patient is on the road to a chronic nephritis. The prognosis of toxic albuminurias, due to prolonged use of certain remedies, is not well established. Cyclic or intermittent albuminuria, appearing during the day and disappearing during the night, oscillating with the toxicity of the urine, is not necessarily a morbid entity, although variations occur in permanent and chronic forms.

Albuminuria minima may denote a slight autointoxication, the relic of an infectious nephritis, or it may signify the insidious beginning of chronic Bright's disease. The light and chronic forms of autointoxication, as a rule, control and influence the albuminurias that they cause; the acute nephritis induced by an acute autointoxication may follow its course independently of its cause. The albuminuria of the mother may be transmitted to the child. Albuminuria with diabetes denotes nephritis, and as the sugar diminishes, the gravity of the prognosis increases with the increase of the albumin.

There are two series of causes of albuminuria,—infection and intoxication. If these causes are intense from the start, the albuminuria may result in the rapid disorganisation of the kidney. If they are of moderate form, albuminuria may exist without provoking infection of the kidney and cease with the disappearance of the cause under favoring conditions.

---

### LACTATE OF STRONTIUM IN KIDNEY DISEASE.

BRONOWSKI (*Times and Register*, December 19, 1896,) tried lactate of strontium in ten cases of kidney disease, three of acute parenchymatous, six mixed and one interstitial nephritis. Six doses of fifteen grains were given daily and were well borne. In all cases the volume of urine increased and its specific gravity fell. The effect began on the second or third day, was most marked on the sixth and seventh days and continued two or three days after the drug was discontinued. The action was most marked in acute cases. The albumin disappeared entirely in the acute cases. The amount of etherial sulphates in the urine was unchanged; neither was there any constant change in the pulse or blood pressure. Bronowski concludes that strontium lactate is a pure diuretic and is more valuable than any other remedy in the treatment of acute inflammatory conditions of the kidney.

---

### GONOCOCCUS PYEMIA.

DR. E. FINGER (*Wiener Wochenschrift*, 1896, p. 248,) states that complications of gonorrhea, such as orchitis, tenosynovitis, bursitis, periostitis, endocarditis and pleuritis, are caused by gonococci carried to these parts from the point of infection. The gonococcus rarely excites superficial inflammation of the mucous membranes; more frequently it causes metastatic inflammation and suppuration, associated with other pathogenic cocci, such as pyogenic, strepto- and staphylococci. It has been shown that the morbid processes produced by gonococci are not unlike, in some respects, to those caused by pus cocci. The pus cocci rapidly permeate the tissues and cause rapid breaking down of the same; the gonococcus is less active and follows the parts of least resistance through the tissues and lacunæ of the epithelium and connective tissues. The gonococcus causes purulent inflammation, with the early formation of abundant granulation tissue. The gonorrheic process forms connective tissue and scars, as in the urethral stricture; in the prostate it destroys the gland, it thickens the suprarenals and causes anchylosis of joints. The gonococcus is less energetic and is more easily destroyed than the pus coccus. The lesions produced by the gonorrheic virus heal more readily than those caused by the pus coccus.—*University Medical Magazine.*

---

### THE RADICAL TREATMENT OF PROSTATIC ENLARGEMENT BY PROSTATECTOMY.

SAMUEL ALEXANDER (*Medical Record*, December 12, 1897,) says whenever the enlarged prostate interferes with the functions of the bladder sufficiently to prohibit urination and to weaken expulsive power, treatment becomes necessary. Delay is then dangerous, menacing both the bladder and the kidneys. There are two methods of treatment, either habitual and life-long catheterisation or removal of the obstruction. If neither of these procedures are possible, an artificial channel for urination must be established and maintained. Operative treatment is not indicated if the obstruction is not great and the power of the bladder is fair; if there is not an excessive amount of residual urine; if catheterisation is easy and painless and cystitis is controlled.

There are many persons afflicted with prostatic enlargement who live in a fair degree of comfort for a long time, often the remaining years of their lives, by more or less frequent catheterism and following a proper course of treatment. There are many others who grow steadily worse in consequence or in spite of catheterism. There are others who have a peculiar immunity from infection, who disregard all rules of cleanliness and antisepsis, and pass along for many years without infection or injury therefrom. If the disease becomes rebellious and palliative treatment is evidently failing, radical treatment should be employed before it is too late. Conditions demanding operative treatment: (1) Complete, or nearly complete, obstruction, making the patient entirely dependent upon the catheter. (2) When there is marked and continuous vesical irritability. (3) When there is a gradual failure of the expulsive force of the bladder, indicated by increasing amount of residual urine. (4) When catheterism becomes more and more difficult and is frequently followed by hemorrhages. (5) When catheterism causes persistent or frequent attacks of cystitis. (6) When there is long-continued cystitis from any cause. (7) When the patient cannot, or will not, use the catheter properly. Operative treatment should follow catheterism before the bladder or the kidneys are hopelessly damaged.

Operations for permanent relief should result in the thorough and prompt removal of all obstructions, with little damage to the mucous surfaces and with efficient drainage of the bladder established. The radical operation consists in the complete removal of all portions of the enlarged gland.

The operation proposed by McGill, Belfield, Nicoll and Alexander fulfill these indications. In the McGill operation the bladder is opened above the pubes and the mucous membrane covering the projecting portions is cut through by scissors and the obstructing portions removed by combined digital enucleation and cutting forceps. Fuller makes a small opening and enucleates with his finger. McGill drains through the suprapubic opening ; Fuller, Keyes and Belfield through the perineum. Belfield was the first to employ the combined suprapubic and perineal operation. Alexander's method : the bladder is distended by eight or ten ounces of antiseptic solution and exposed by vertical incision between the recti muscles and the bladder is retracted by two sutures ; the incision is made between them sufficiently large to receive two fingers. The patient is then placed in the lithotomic posture, the membranous urethra opened by a median perineal incision and thoroughly cut to the apex of the prostate. With two fingers of the left hand passed into the bladder, through the suprapubic opening, the gland is pressed into the perineum. The forefinger of the right hand is pressed into the perineal opening, breaks through the inferior surface of the prostatic capsule, shells out the enlarged gland by digital dissection. During this work the gland may be drawn down by forceps. The irrigation is done by tubes through these openings. The suprapubic tube is removed the fourth day, the perineal the seventh day.

Dr. Alexander claims the following advantages for his operation: (1) The entire gland is enucleated thoroughly and promptly removed. (2) The mucous membrane of the bladder and prostatic urethra is uninjured and the danger from septic absorption is lessened. (3) Hemorrhage is reduced to a minimum. (4) The most efficient and thorough drainage is secured. (5) The time required to perform this operation is comparatively short.

